# VAST (Volume Annotation and Segmentation Tool): Efficient Manual and Semi-Automatic Labeling of Large 3D Image Stacks

**DOI:** 10.3389/fncir.2018.00088

**Published:** 2018-10-16

**Authors:** Daniel R. Berger, H. Sebastian Seung, Jeff W. Lichtman

**Affiliations:** ^1^Department of Molecular and Cellular Biology, Harvard University, Cambridge, MA, United States; ^2^Computer Science Department, Princeton Neuroscience Institute, Princeton University, Princeton, NJ, United States

**Keywords:** connectomics, segmentation, visualization, serial section electron microscopy, CLEM, proofreading, TrakEM2, voxel

## Abstract

Recent developments in serial-section electron microscopy allow the efficient generation of very large image data sets but analyzing such data poses challenges for software tools. Here we introduce Volume Annotation and Segmentation Tool (VAST), a freely available utility program for generating and editing annotations and segmentations of large volumetric image (voxel) data sets. It provides a simple yet powerful user interface for real-time exploration and analysis of large data sets even in the Petabyte range.

## Introduction

The acquisition of microscopic data is becoming ever faster and more and more automated, leading to the generation of enormous image datasets. At the same time progress in processing speeds and storage capacity of computer hardware enables imaging scientists to work with big data. In neuroscience acquisition of high-resolution volumetric data sets of the nervous system has become routine, with the goal of addressing a number of long-standing questions (Briggman and Bock, 2012; Helmstaedter, 2013; Morgan and Lichtman, 2013, 2017; Plaza et al., 2014; Titze and Genoud, 2016). Projects include descriptions of the entire nervous systems of a variety of animals, for example *Caenorhabditis elegans* (White et al., 1986; Varshney et al., 2011), *Drosophila melanogaster* (Zheng et al., 2017), Zebrafish (Hildebrand et al., 2017); wiring diagrams of specific parts of larger nervous systems, for example mouse retina (Helmstaedter et al., 2011, 2013; Kim et al., 2014; Bae et al., 2018), thalamic nuclei (Morgan et al., 2016), and cortex (Bock et al., 2011; Kasthuri et al., 2015; Lee et al., 2016); function-structure relationships, for example directional selectivity in the retina (Briggman et al., 2011; Kim et al., 2014), detection of visual motion in drosophila (Takemura et al., 2013), learning and plasticity in hippocampus (Mishchenko et al., 2010; Bartol et al., 2015), synapse elimination in the neuromuscular junction (Tapia et al., 2012); and many others.

Several experimental techniques have been introduced to enable processing and imaging such large volumes of tissue with electron microscopy. Among these are the development of advanced techniques for preparing and staining very large pieces of tissue for electron microscopy (McIntyre and Fahy, 2015; Mikula and Denk, 2015), block-face cutting and imaging methods like SBEM/SBF-SEM (Denk and Horstmann, 2004) and FIB-SEM (Knott et al., 2011; Hayworth et al., 2015), automated collection of sections on tape, for example ATUM (Hayworth et al., 2006; Kasthuri et al., 2015), and high-speed imaging techniques like TEMCA (Bock et al., 2011), and the Zeiss mSEM (Eberle et al., 2015).

Because of the wider availability of these methods, analyzing big data sets poses a challenge for a growing number of researchers. Many existing software tools are not well suited for big data nor the wide variety of research questions these data sets allow. Many, if not all, of the software applications for analyzing microscopic image data allow labeling of cellular or subcellular constituents of the volumetric tissue. This fundamental process is called segmentation and can be done in several ways including: (1) Bread crumbs/Seeds; Markers are placed inside objects to identify their location from one section to the next, (2) Skeletonization; If objects have a tree- or graph-like structure, they can be described by a set of nodes that are connected by straight lines called edges, (3) Outlining; Objects of arbitrary shape can be delineated by their outlines in each section to create the surface of a 3-dimensional object, (4) Voxel painting; Objects of arbitrary shape can be labeled by filling in their area in each section to create a volume.

As the number of biological laboratories analyzing large image data sets is increasing, it is the purview of computer scientists to create the (segmentation) tools for analyzing such data sets. Taking into consideration the diverse range of potential applications from 3D microscopy, an ideal segmentation tool would have the following features:

• **Usability.** Easy to set up and use, without complicated dependencies on external libraries and packages; accessible documentation.
• **Scale and speed.** Ability to work with Petabyte-sized data sets interactively, including data sets stored in online databases.
• **Interactivity.** Easy import and export functions, to enable interactivity with other programs (for example for image stack alignment, data analysis and 3D rendering).
• **Versatility:** Full freedom to label any 3D object in the dataset, for example to generate flexible ground truth for automated segmentation by artificial neural networks.
• **Organization** of labeled objects to represent object classes and parts.
• **Flexible visualization:** Multi-layer image stack overlays and configurable color channels for light microscopy (LM) and correlated light and electron microscopy (CLEM) applications, selective display and highlighting of relevant objects and semantic object groups.
• **Automation:** Ability to make use of automatic segmentations if available, to help manual segmentation and/or as a basis for manual proof-reading.
• **Data privacy:** Full control over who can access the data set.
• **Extensibility, scripting:** Users may want to write their own scripts that exchange data with the segmentation program, for example for custom data analysis.

**Table 1** shows a comparison of features of several popular programs for EM stack segmentation. Many tools were originally made for smaller data sets and require the complete data to be loaded in memory, which is not feasible for large image stacks, though for some programs workaround exist; these include ITK-SNAP (Yushkevich et al., 2006), trakEM2 (Cardona et al., 2012), Reconstruct (Fiala, 2005), which loads two complete sections at a time, ilastik (Sommer et al., 2011), and IMOD (Kremer et al., 1996). Other tools are limited to be used only by their respective developers, like Eyewire (Marx, 2013; Kim et al., 2014; Bae et al., 2018), and/or are specialized for skeleton tracing, like Catmaid (Saalfeld et al., 2009), Knossos (Helmstaedter et al., 2011), and WebKnossos (Boergens et al., 2017), or for splitting and merging for proof-reading of automatic segmentations like Raveler (Chklovskii et al., 2010; Takemura et al., 2013) and its successor NeuTu^1^. The professional tools Imaris (Bitplane Inc.) and Amira-Avizo (Thermo Fisher Scientific) have work-arounds to use large data sets that cannot be fully loaded into memory. However these latter programs appear to have only rudimentary tools for manual segmentation and are not specialized to do voxel painting or proof-reading of automatic segmentations. Neurolucida (MBF Bioscience) is a specific tool for analyzing light microscopy data and appears to be RAM-size limited. Neurolucida 360 does support large datasets beyond the machine’s RAM limit, however it is still focused on light microscopy applications.

**Table 1:** Feature comparison of popular 3D EM analysis tools.

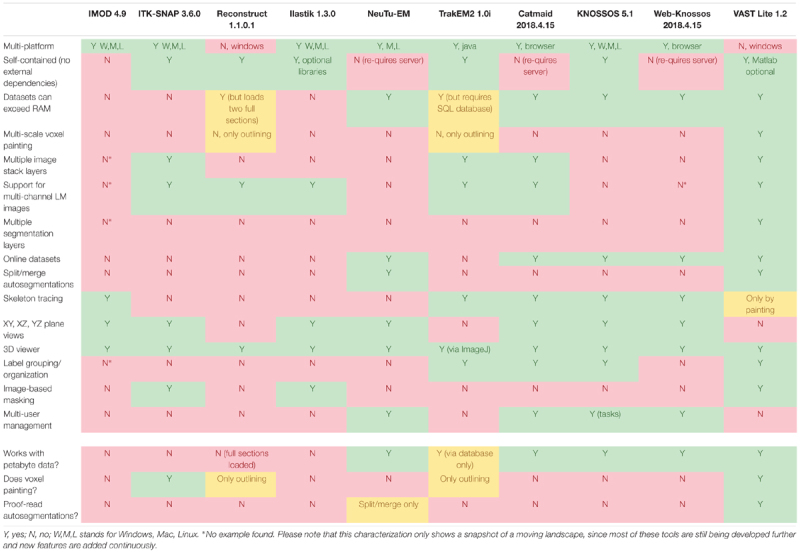

To address the requirements of an ideal segmentation tool and to supersede the functionality of available segmentation tools, we built VAST, a lightweight, freely available utility program for manual annotation and segmentation of large volumetric image (voxel) data sets even in the Petabyte range. VAST is written in C++ with Direct3D graphics for optimal performance (see **Figure 7** for information about the internal program structure). It provides an intuitive yet powerful user interface for exploring image stacks at interactive speeds, and for labeling structures of interest by voxel painting at multiple resolutions. As **Table 1** shows, VAST solves many of the problems that beset other tools. For ground truth annotation by voxel painting VAST is an excellent choice. In addition, because of the availability of automatically segmented data, we found VAST can reconstruct whole volumes faster than fully manual segmentation (see below). Although this tool was developed for neural circuit analysis of EM datasets, it can load and process any three-dimensional 8- or 24-bit image stack and be used for other applications like multi-color light microscopy, CLEM, video analysis and object tracking. VASTs extensive import and export functions make it easy to integrate it with other applications.

Volume Annotation and Segmentation Tool can open grayscale and RGB image stacks which have been either imported into VAST’s own 3D data file format or are stored locally in image tiles, and it can stream image data from several online sources. Multiple image and segmentation layers of the same dataset can be loaded and displayed together. Segmented objects can be named, grouped and organized in a tree structure, and segmentations and their metadata can be imported and exported. Automatic segmentations can be proof-read with merge and split operations and novel *trans*-layer masking techniques. Custom client programs can exchange data with VAST via a documented API. VAST includes the API client program “VastTools” which runs in Matlab (The Mathworks, Inc.) and provides additional functions for exporting, measuring, and navigating data loaded into VAST.

Volume Annotation and Segmentation Tool has been key for the data analysis for a number of scientific papers and continues to be a versatile tool with growing functionality and an expanding user base (Tomassy et al., 2014; Kasthuri et al., 2015; Ai-Awami et al., 2016; Joesch et al., 2016; Ke et al., 2016; Morgan et al., 2016; Quadrato et al., 2017; Sheu et al., 2017; Zung et al., 2017). Because VAST is designed as a general labeling tool, it is not limited to tracing neurites, but can be used for a large variety of 3D data sets and tasks (see **Figures 5, 6**). This includes working with electron-microscopic, multi-channel light-microscopic, and Micro-CT data sets as well as videos, and annotating arbitrary structures, regions and locations, depending on the user’s needs.

The version of VAST discussed in this paper is VAST Lite 1.2. An earlier version of VAST, which lacked most of the key features reported here, was briefly discussed in the methods section of (Kasthuri et al., 2015). New features implemented since then include: VSVI files (section “Reading Image Files Directly, .VSVI”), VSVR files for Google Brainmaps and Butterfly servers (section “Reading From Online Databases, .VSVR”), collect tool (section “Organization of Segments in Hierarchies”), working with multiple image stack and segmentation stack layers (section “Working With Multiple Image and Segmentation Stack Layers”), image layer coloring and blending (**Figure 3**), filling tool (section “Manual Segmentation by Drawing and Filling”), *trans*-layer masking (section “Working With Automatic Segmentations” and **Figure 4**), the 3D viewer (section “The Integrated 3D Viewer” and **Figure 5**), and the API and VastTools (section “The VAST API and VastTools”). At the time of writing of this manuscript, the current version of VAST can be downloaded at: https://software.rc.fas.harvard.edu/lichtman/vast/.

## The Vast User Interface

**FIGURE 1:**
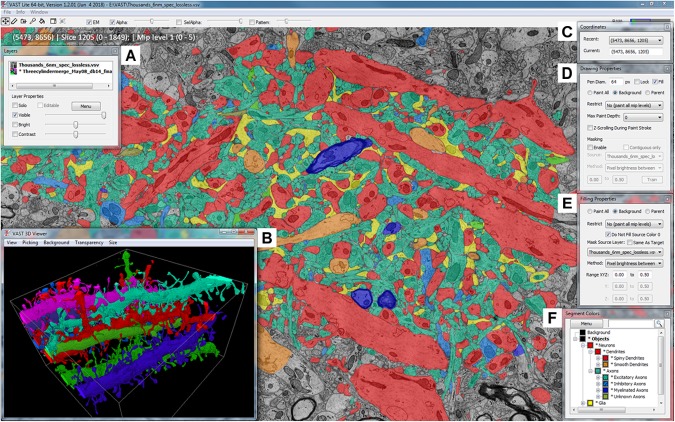
The Volume Annotation and Segmentation Tool (VAST) user interface. The main window of VAST shows an EM dataset with a manual segmentation layer as transparent overlay in which segments are colored by type (colors of collapsed folders in the “Segment Colors” tool window). The “3D Viewer” window **(B)** shows spiny dendrites in the area in individual colors. The tool windows **A** and **C–F** are explained further in section 2 of the main text.

**FIGURE 2:**
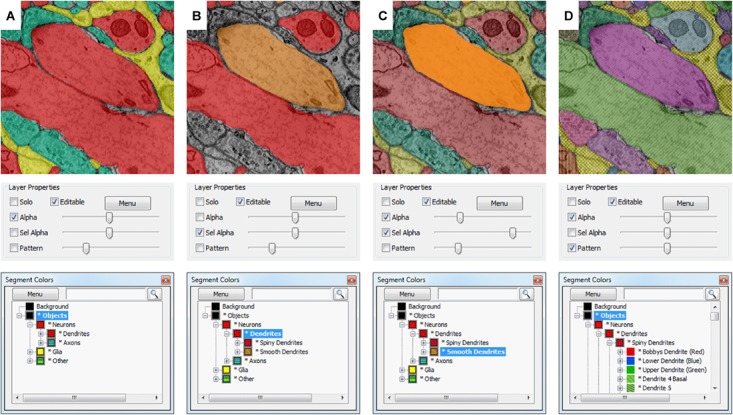
Selective segmentation display. Which segments in a segmentation layer are shown in what color depends on the selection and folder collapse state of the segment hierarchy. The **top** shows the appearance in the VAST main window; the **middle** shows the transparency and pattern settings of the segmentation layer in the “Layers” tool window, and the **bottom** shows the “Segmentation Colors” tool window. **(A)** All object type folders are closed, so all objects are shown in colors depending on their identity (Dendrite, Axon, Glia, Other). Patterns are disabled. **(B)** By enabling “Sel Alpha” for selective opacity control of the selected branch, disabling Alpha and selecting the “Dendrites” folder, now only dendrite segments are shown and colored depending on their subtype (spiny or smooth). **(C)** When “Alpha” and “Sel Alpha” are both enabled, the opacity of the selected subfolder and all other segments can be controlled separately. In this example, the segments in the “Smooth Dendrites” folder are given a higher opacity with the “Sel Alpha” slider to highlight them. **(D)** The folders in the segmentation hierarchy are opened such that all neurites and glial branches in the segmentation are shown with individual colors. Here patterns are enabled, showing all segments with their individual patterns. The strength of the patterning can be controlled with the “Pattern” slider.

Volume Annotation and Segmentation Tool’s user interface is based on familiar Windows controls and is optimized for efficient use of pen tablets for fast and accurate user interaction. The main window of VAST (**Figure 1**) shows a 2D section of the loaded dataset(s) and has several floating tool windows which can be moved and resized. The tool bar of the main window provides quick access to the different editing tools, as well as a switch to hide all image layers (EM), and sliders to control the opacity of the selected segmentation layer (Alpha) and, if enabled, separately for the segment or group of segments selected in the ‘Segment Colors’ tool window (SelAlpha). To increase the number of distinguishable segment colors beyond RGB, VAST can combine two 24-bit RGB colors with one of 16 patterns to reach a color space of almost 52 bits. The strength of the patterns can be controlled with the ‘Pattern’ slider. **Figure 2** shows examples of different settings of Alpha, SelAlpha, and Pattern. Further options for color correction and blending of individual layers are provided in the ‘Layers’ tool window (**Figure 1A**).

The ‘Coordinates’ tool window (**Figure 1C**) shows the current volume coordinates (center point of the main window) which can be copied from and pasted into the text field to store and go to particular coordinates in the stack. Its drop-down menu lists coordinates that were recently visited.

The ‘Drawing Properties’ tool window (**Figure 1D**) contains the parameters relevant for the pen tool, including settings for the optional ‘masking’ mode. This mode can be used to constrain manual painting by an automatic segmentation result, which can lead to an increase of accuracy and speed of manual tracing, even if the automatic segmentation has errors.

Equivalently, the ‘Filling Properties’ tool window (**Figure 1E**) contains the parameters relevant for the filling tool. Filling can also use masks derived from colored regions in a separate source layer, which allows for efficient proof-reading of automatic segmentations (split and merge operations).

The ‘Segment Colors’ tool window (**Figure 1F**) lists all segments used in the selected segmentation layer, with their color and label, in a tree folder structure which represents grouping and parts/subparts relationships of segments. Collapsing and expanding these folders determines the display colors in the main window – segments in collapsed folders will be shown in the folder color (**Figure 2**). The search field at the top of the tool window can be used to find segments by (part of) their label text or their internal ID. The ‘Menu’ button leads to a context window with many more functions to edit segment properties and the tree hierarchy.

All tool windows are listed under ‘Window’ in the main menu; this also includes a window with control buttons to be used with touch screens, a window showing all keyboard shortcuts, and the options window for the ‘Remote Control API server’ to link to external programs via TCP/IP.

## Importing Image Stacks

Volume Annotation and Segmentation Tool can access image data from three types of sources: (1) image stacks which have been imported into VASTs own data file format (“VAST Volume”, .VSV/.VSVOL), (2) image data sets stored as image files, accessed via a .VSVI descriptor file (“VAST Volume of Images”), and (3) data sets stored online, accessed via a .VSVR descriptor file (“VAST Volume of Remote data”). Segmentation data can be imported from image files representing segment IDs, and optionally a metadata description file, into VASTs segmentation file format (.VSS/.VSSEG). The image file formats from which VAST can import are listed in **Table 2**.

**Table 2:** Available data and file formats for importing and exporting in Volume Annotation and Segmentation Tool (VAST) and VastTools.

Importing	To	Data formats	File formats
	EM/LM image stack files, .VSV/.VSVOL	8 bit, 24 bit images	.png, .tif, .bmp, .jpg
	Segmentation stack files, .VSS/.VSSEG	16 bit IDs as images	.png, .tif, .txt for metadata
	.VSVI image tiles	RGB, graylevel images	.png, .tif, .bmp, .jpg
			
**Exporting**	**To**	**Data formats**	**File formats**
VAST	EM/LM image stacks	8 bit, 24 bit images	.png, .tif, .raw
	Segmentation stacks	16 bit IDs as images	.png, .tif, .raw
	Screenshot stacks	24 bit (RGB) images	.png, .tif, .raw
	3D viewer screenshots	24 bit (RGB) image	.png, .tif, .bmp
	Segmentation metadata	Text file	.txt
VastTools.m	3D object meshes	Triangle mesh	.obj/.mtl
	Isosurface shells	Triangle mesh	.obj/.mtl
	3D particle clouds	Triangle mesh	.obj/.mtl
	3D boxes	Triangle mesh, texture	.obj/.mtl, .png
	3D scale bars	Triangle mesh	.obj/.mtl
	Projection images	24 bit (RGB) image	.png, .tif, .bmp, .jpg
	Surface measurements	Text file	.txt
	Volume measurements	Text file	.txt
	Particle metadata	Text file	.txt


### VAST Image Data Files, .VSV/.VSVOL

These files store a complete image stack together with a resolution pyramid of lower-resolution versions of the same image data [“mipmaps”, (Williams, 1983)]. During importing, the image data is reordered as 16 pixel × 16 pixel × 16 pixel cubes in optional Z-order and packed with lossless or lossy compression. Mipmaps are precomputed. A file-internal tree of pointer blocks is generated which allows VAST to access arbitrary regions with minimal overhead.

Keeping the image data of one data set in a single file has the advantage that it can only be copied as a whole, making storage and distribution of data sets simpler. Also, the file system does not have to handle thousands or millions of image files. However, this comes at the disadvantage that images in the dataset cannot be modified or the stack extended without regenerating the data file. Also, the importing procedure is impractical for large datasets, not only because the target file can get unwieldy, but also because importing can take a very long time since processing cannot be easily parallelized. For example, importing the ∼6.8 teravoxel data set of (Morgan et al., 2016) took around 30 days on a single computer, with disk and network I/O being the largest bottleneck. Therefore, for large datasets (exceeding one terabyte), we typically keep the image data in individual image tile files which VAST can load directly.

### Reading Image Files Directly, .VSVI

Image stacks can be kept as a collection of image files (.PNG, .TIFF, or .JPG) with a descriptor file for VAST (.VSVI, “VAST Volume of Images”). VAST can then load and cache specific regions directly from the images. The image files have to be stored in a regular directory structure, and reduced-resolution images (mipmaps) have to be precomputed and stored as separate files. Then, a .VSVI file for VAST is prepared. This is a text file following the JSON syntax which specifies the naming scheme and storage location of the image tiles, as well as other metadata for the data set. The .VSVI file can be opened in VAST, which then loads regions of the dataset from the image files as requested.

### Reading From Online Databases, .VSVR

Volume Annotation and Segmentation Tool can also stream in remote data from online databases. Some data sets are too large to be stored locally, and/or they reside on a server which is accessible via HTTP. VAST can load data from such sources dynamically by requesting parts of the data set from the server. Currently, protocols for openconnecto.me, neurodata.io, Harvard Butterfly servers, and Google Brainmaps are supported. The specification of the source address, the data request protocol, and additional metadata is stored in a .VSVR file (also a JSON text file). Once such a file is opened, VAST will request and stream in image data from the server dynamically. In the case of Google Brainmaps, VAST will first negotiate access rights with the server through the OAuth2 protocol (including user login in a browser window). VAST always caches the image data locally to optimize speed and minimize the network load. Several .VSVR files linking to existing online datasets are included in the VAST supplementary package (see section “The VAST API and VastTools”).

### Importing Segmentations, .VSS/.VSSEG

Similar to.VSV files described above, VAST stores segmentations in single files with extension .VSS or .VSSEG. These files contain the voxel data in 16 pixel × 16 pixel × 16 pixel cubes, including mipmaps, as well as the metadata for the segments. To allow segmentation files to store arbitrary subregions of a large data set and to make arbitrary extension of those regions possible as users continue to trace, a tree of file-internal pointer blocks (16 × 16 × 16 pointers per block/tree node) is maintained which references the storage location of different segmentation image blocks within the segmentation file. Pointer blocks are also cached in memory when the segmentation file is opened in VAST for optimal file access speed. Selective storage of subregions of the dataset keeps file sizes small if sparse segmentations are generated on very large image stacks.

## Organizing Data in Vast

In VAST, image and segmentation data can have attributes in three separate categories; multiple resolutions, semantic hierarchies of segments, and multiple layers showing different data from the same location. The following sections discuss these possibilities.

### Multiresolution Voxel Representation of Image and Segmentation Stacks

Different from many other segmentation tools for large image stacks, VAST uses voxels not only to represent image stacks, but also segmentations. A voxel is a three-dimensional image element, equivalent to the two-dimensional pixel. Analogous to the brightness or color value of a voxel in a microscopic image stack, in a segmentation layer in VAST each voxel stores one ID (segment identifier number). When VAST displays segmentation data, these IDs are translated to colors based on a metadata table.

Voxel representations of segmentations are typically more memory-intensive than vectorized outlines (Fiala, 2005) or ‘area lists’ (Cardona et al., 2012), because an ID value has to be stored for each voxel rather than a set of coordinate points and edges around the perimeter of the segment region. However, a segmentation stored in voxels has a more direct relationship to the image stack it is based on, can directly represent segmentation data produced by machine learning algorithms which is typically also voxel-based, and allows for voxel-by-voxel masking and filling.

While scaling of vector data is straight-forward in computer graphics, voxel data is more difficult to handle. In VAST, image and segmentation stacks use mipmaps to limit the amount of voxel data which must be handled at one time. Mipmaps are lower-resolution versions of the original images. VAST uses powers-of-two XY mipmaps, which means that for example for original images of 4096 pixel × 4096 pixel, there will also be versions with 2048 pixel × 2048 pixel, 1024 pixel × 1024 pixel, 512 pixel × 512 pixel, and 256 pixel × 256 pixel available. Because the number of pixels on a computer screen is limited, the data which has to be loaded and displayed to fill the screen is always limited by the extent of the display area and the on-screen resolution. Like Google Maps, by loading just the part of the data set at the resolution necessary for the current zoom level and view region, VAST loads and caches data for display from the center of the view area outwards as needed.

Volume Annotation and Segmentation Tool implements LRU (least-recently-used) memory caches for image and segmentation data. LRU caches remove the least recently used image blocks first when memory runs low. Individual cache blocks can be locked and marked as modified. For segmentation data, VAST preferentially removes unmodified cache blocks. Modified cache blocks cannot be deleted, so disk buffering is used when the memory cache overflows with modified cache blocks.

### Organization of Segments in Hierarchies

Grouping objects into different classes and representing each object as a hierarchy of parts can be an important intermediate step for further analysis of labeled objects in a data set. For example, counting spines or measuring their volume and other morphological properties becomes much easier once each spine is represented as a separate sub-object of its dendrite. In VAST, segments can be arranged in a configurable hierarchy tree which is visualized in the ‘Segment Colors’ tool window. VAST uses the segment hierarchy tree to selectively color and display objects in different sub-branches. Objects in collapsed folders are shown in the folder color, and transparency can be separately controlled for the selected segment and its children versus all other segments, so that users can hide or highlight semantic groups of objects (see **Figure 2**). The same hierarchy is also used for selective exporting, and for operations on the segmentation layer like drag and drop, deleting and welding of branches. The tree structure can be exported or accessed via the API together with the rest of the segmentation metadata (segment IDs, labels, anchor points, bounding boxes) and analyzed externally.

Volume Annotation and Segmentation Tool provides a “Collect” tool which can be used to collect (translocate) segments which are clicked in the 2D view into the selected folder in the segment hierarchy. This can be used to quickly classify segments into different classes represented by different folders in the hierarchy. Since each segment stores an anchor point and a label text, segments can also be used as bookmarks to annotate and store locations of interest. The paint color can be used as a visual marker.

### Working With Multiple Image and Segmentation Stack Layers

**FIGURE 3:**
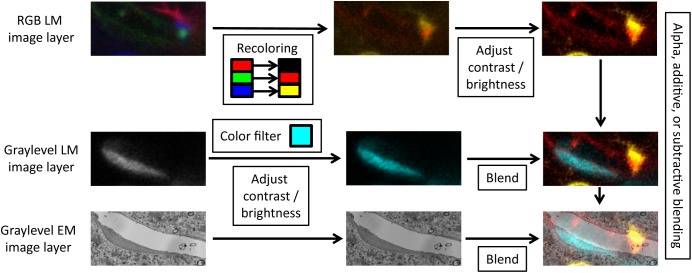
Layer blending. For each image layer, VAST can apply color filters, recolor channels of RGB images, adjust contrast and brightness, and blend the layers with different modes.

Volume Annotation and Segmentation Tool can load multiple image and segmentation layers of the same dataset at the same time, visualize them together in 2D and 3D, and use the data in one layer to guide labeling in another (*trans*-layer masking). Both 8-bit graylevel and 24-bit RGB images are supported for image layers. Segmentation layers are currently limited to 16-bit values per pixel (65535 segments maximally). Since VAST can load multiple graylevel and RGB image stack layers, it can also be used to visualize and annotate multi-channel light-microscopic image stacks. VAST can assign an arbitrary color to each channel, and filter and blend the layers in several ways (see **Figure 3**). Using masking, 3D models can be traced semi-automatically from optical image data. For combined light and electron microscopy (CLEM) data sets, once aligned, EM and LM image stacks can be loaded together and superimposed, for example to allow for easy identification of structures in the electron-microscopic images which are fluorescently labeled in the light-microscopic image stack.

Volume Annotation and Segmentation Tool can also load more than one segmentation layer at once. This can be useful if single pixels should be given more than one ID. For example, one segmentation layer can be used to trace out axons and dendrites, and a second one is used to trace organelles (mitochondria, vesicles, synaptic contacts; see **Figures 6D–F**). The overlap of the labels in different layers can then be used by an external script to analyze which organelles are in which axons and dendrites, and which axons and dendrites are connected with synapses and where. This method was used to compute the synaptic connectivity in (Kasthuri et al., 2015).

Another application is to load different parts of a segmentation and display them together, if those parts are stored in separate files, for example if several people work on segmenting different parts of the same data set.

## Generating and Editing Segmentations

### Manual Segmentation by Drawing and Filling

Volume Annotation and Segmentation Tool provides a pen tool with adjustable tooltip size and a 3D fill tool for manual editing. They can be accessed via the pencil and the paint bucket button in the toolbar. A pipette tool is also provided which selects the segment clicked in the 2D view for painting.

In VAST, users always edit the segmentation at the currently viewed mipmap resolution. A segmentation layer can thus contain segmented objects at different resolutions, and VAST automatically combines the information from different mip levels as the user moves and zooms through the data set. This allows for voxel painting on very large image stacks. Voxel painting speed in VAST is independent of the zoom level, allowing users to paint very large regions (gross morphology, cell bodies) at low resolution as instantly as fine axonal processes at high resolution. To our knowledge VAST is the first and only application in existence which provides this functionality.

Editing in VAST is non-destructive in the sense that the source segmentation files are not changed unless the user saves changes back to the opened file explicitly. All changes are kept in VASTs cache system until the user saves them or discards them by exiting the program.

The parameters of the pen tool are accessible in the “Drawing Properties” tool window (see **Figure 1D**). The diameter of the tooltip can be set to specific values and locked. If “Fill” is enabled in the “Drawing Properties” tool window, VAST will automatically fill empty closed contours as they are drawn. Users can choose to paint on all voxels or to restrict painting to only empty voxels or to voxels of the direct parent segment of the selected segment in the hierarchy. Painting and filling can be restricted to a specified mip level, so that a required resolution can be guaranteed. If the “Max Paint Depth” option is set to a nonzero value, VAST will look in the Z-stack for voxels with the paint color (selected segment ID) at the same XY coordinates in neighboring sections and fill the gap in Z with the paint color, up to the specified distance. This can be used to speed up rough manual tracing, for example by setting “Max Paint Depth” to +-8 and painting outlines of the object only in every eighth section. VAST will fill in the vertical overlap between painted outlines in the seven sections which were skipped. Users can then check and correct the segmentation where needed to refine the object shape.

When users hold down the “Delete” key, which can be mapped to buttons on the pen of a tablet, the pen will erase instead of painting. Holding down the “Shift” key will allow users to pick colors from the 2D view. As long as the “Control” key is held down, users can pan the view with the pen or mouse instead of painting. Holding down the “Tab” key allows for quick changes of the pen tooltip size. These modifiers allow for rapid and intuitive access to the different functions needed during manual painting.

The parameters of the “Fill” tool can be set in the “Filling Properties” tool window (**Figure 1E**). Some of the options are (linked) duplicates of the same options in the “Drawing Properties” tool window described above. The fill tool can be used to recolor complete connected components of segments with a single click, but it shows its real power when used in combination with *trans*-layer masking, as described below.

### Working With Automatic Segmentations

While VAST does not generate automatic segmentations itself, it can be used to proof-read automatic segmentations generated elsewhere. Automatic segmentations can be loaded into VAST in two fundamentally different ways. First, they can be loaded as an image layer and used to guide manual segmentation in a separate segmentation layer. This allows users to generate a separate proof-read copy of the segmentation data. Second, if the automatic segmentation does not exceed the 16-bit limit for number of segments (65,535), it can be loaded as a segmentation layer itself and then edited by the user with split and merge operations as well as fully arbitrary manual corrections. The VAST download page contains a link to Youtube tutorial videos illustrating the proofreading process (specifically^2^^,^^3^).

#### Using Automatic Segmentations to Guide Manual Painting

**FIGURE 4:**
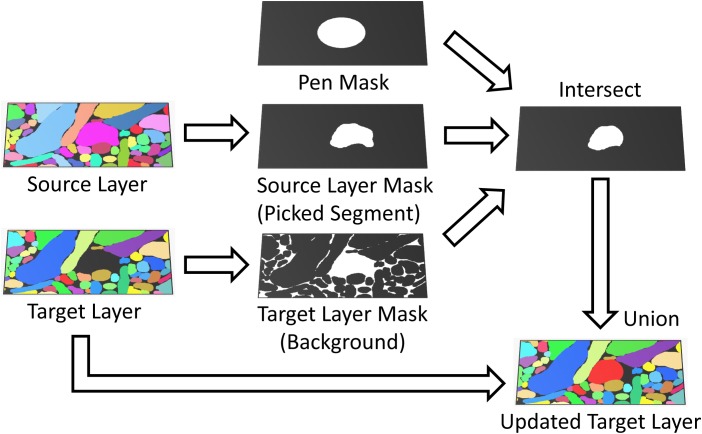
Masked painting. During painting, VAST intersects a mask of the pen tooltip (Pen Mask) with a mask derived from the target layer (Paint All, Background or Parent) and optionally, if “Masking” is enabled, a mask derived from an additional source layer (Brightness/Color range or Picked Segment) to constrain which voxels in the target layer are painted. This can for example be used to guide manual painting in the target layer by an automatic segmentation in the source layer.

Volume Annotation and Segmentation Tool can use two types of automatic segmentation results to guide manual painting: boundary (probability) maps, in which each voxel is assigned its probability to be located on a boundary between objects, and candidate segmentations, in which each voxel stores an object ID. In both cases the automatic segmentation is loaded as an image layer (source layer) and the “Masking” feature is used to constrain the painting area in a separate segmentation layer (target layer) based on information from the source layer (see **Figure 4**). In this mode, parts of the automatic segmentation can be transferred from the source to the target layer by the user, keeping the “raw” source and the “proof-read” target segmentations strictly separate.

To use a boundary probability map for this process, it is selected as the source layer, and VAST’s “Masking” mode is set up to restrict painting to contiguous regions of “interior” voxels only (excluding the boundaries) by setting an appropriate source layer pixel value range. Either the pen or the fill tool can then be used to create segments with boundaries defined by the boundary probability map.

If the automatic segmentation in the source layer provides candidate objects or supervoxels instead, masking can be set up so that for each pen stroke or fill operation, the color in the source layer is picked and painting of the new segment in the target layer is constrained to the region in which the source layer has the picked color (as shown in **Figure 4**). In this mode, single segments can be copied from the source to the target layer with the pen and fill tools, which allows for correction of split and merge errors. Another strategy is described in section “Correcting Automatic Segmentations in VAST”. VAST supports 8-bit and 24-bit image layers, which restricts the number of representable distinct objects to a maximum of 16,777,215. However, data sets with more segments can be loaded as well, though only 24 bits of their object IDs will be available in VAST. This can lead to mergers of neighboring objects in rare cases, if the originally different IDs are represented by the same 24-bit number. Those mergers can however be corrected easily by the user. In this manner segmentations with virtually unlimited numbers of segments can be used to guide the generation of a proof-read segmentation. Care should be taken during importing such that segments with IDs of multiples of 2^24^ are not mapped to 0 and disappear.

#### Correcting Automatic Segmentations in VAST

When an automatic segmentation is loaded into VAST as a segmentation layer, the user has full freedom to manipulate the segments by painting or erasing. Split errors (cases in which a single object consists of several parts in the segmentation) can be corrected non-destructively by collecting all parts of the object into a folder using the ‘Collect’ tool. If necessary, all segments of a folder can be “welded” to a single segment. Merge errors (several actual objects appear as a single object in the segmentation) are more difficult to correct because the user must define where within the merged object the boundaries should be. To correct mergers, the user can paint over part of a segment with a different segment. For this, VAST’s “Parent” mode can be used, which restricts painting to the immediate parent of the current paint color in the segment hierarchy. One side of the split boundary is relabeled in a different segment color by painting, and the remaining part of the branch which should be split off is recolored by filling. Alternatively, the user can split an object into separate connected components by erasing the segment at the point of connection to disconnect the parts, and then use the fill tool to change the segment ID of one of the connected components. Afterwards the boundary where the split was performed can be cleaned up by manual painting.

On-demand automatic segmentation of very large data sets is in principle possible if the segmentation image stack is loaded from a web server and the segmentation is done on the server side. VAST would request parts of the automatic segmentation from the server, which would do the on-demand computation and send the result to VAST. This approach could for example be used to trace axons quickly over very large distances without requiring a complete automatic segmentation of the whole data set.

### Multi-User Segmentation, Splitting and Merging of Segmentations

Even though VAST is not a client-server solution where multiple users can edit a single segmentation at the same time, by requesting data from and committing changes to a central data server, it is possible to have several scientists work on the same dataset and combine the results. VAST provides a merge function by which several .VSS segmentation files can be merged into one, with options to define voxel overwrite and segment ID renumbering rules. When merging a ‘source’ segmentation onto a ‘target’ segmentation, conflicts are resolved on a voxel-by-voxel basis. The user can decide whether nonzero target voxels can be overwritten by merged-in source data (source precedence) or are write protected (allowing only empty target voxels to be written; target precedence). VAST can also export and import segmentations as image stacks with metadata, allowing for more complicated merge procedures done externally. Also when importing segmentation images onto an existing segmentation, either source or target precedence can be applied.

Parts of segmentation files can be recombined by using branch deleting or branch exporting and merging. A selected segment or branch of segments (selected segment and child tree in the segmentation hierarchy) can be saved to a separate .VSS file using “File/Save Segmentation As Special …” and subsequently merged with a target segmentation file. Alternatively, the “Delete Segment + Subtree” function from the context menu of the “Segment Colors” tool window can be used to delete all unwanted segments, and the result can be saved to a separate .VSS file and then processed further.

## Data Visualization, Exporting, and Analysis

### The Integrated 3D Viewer

**FIGURE 5:**
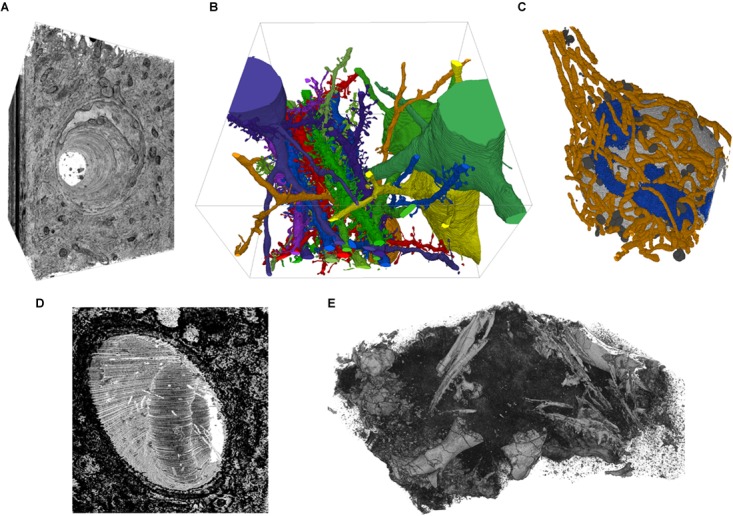
Example screenshots of the 3D viewer. **(A)** Capillary running through cortex, rendered from EM image stack. **(B)** Segmented spiny dendrites and cell bodies (fully manual segmentation). **(C)** Organelles in a neuron soma; mitochondria in orange, Golgi apparatus in blue, lysosomes dark gray, nucleus light gray (fully manual segmentation). **(D)** Erythrocyte in a capillary in LGN rendered from an EM image stack. **(E)** Micro-CT scan of a fossil specimen of *Paleothyris acadiana* (Museum of Comparative Zoology, Harvard). Data in **A–C** from (Kasthuri et al., 2015); **D** from (Morgan et al., 2016); **E** with kind permission of S. Pierce, Museum of Comparative Zoology, Harvard.

The 3D viewer in VAST can be used to inspect and visualize image and segmentation stacks. It makes use of a volumetric texture with transparency rather than surface meshes to display voxel data in its native format. Because the 3D textures are retrieved from the 2D view, the same coloring and blending options are available, and image and segmentation stacks can be visualized together. The transparency of the 3D textures can either be derived from the pixel brightness or set to opaque to show a full cube. The view can be rotated and zoomed and screenshot images can be exported. The user can also click on objects in the 3D viewer to set the 2D window to the same location. **Figure 5** shows a selection of examples rendered with the 3D viewer.

### Image Stack Exporting

For further processing and visualization of voxel data in other applications, VAST includes functions to write image and segmentation data back to stacks of image files. The image stack export dialog can be found under “File/Export …” in the main menu. VAST can export image stacks of data in single image layers, segmentation layer data (images encoding IDs in blue and green color channels), and the composited 2D view (“screenshots”), of a definable sub-region of the loaded data set, at a definable mipmap resolution. Large sections can be exported as a mosaic of image tiles. Metadata for segmentation layers can be exported to text files using the “Save Segmentation Metadata …” function in the menu of the “Segment Colors” tool window. VAST provides a Matlab script to parse these text files. **Table 2** lists the image formats which are available for image stack exporting.

### The VAST API and VastTools

Volume Annotation and Segmentation Tool includes an API which can be accessed from client programs through the TCP/IP network protocol, either locally on the same computer or remotely through a network connection. Once the TCP/IP port is enabled in VAST, client programs can connect and send API commands to VAST to exchange data. The protocol for the API and all API functions are documented in the user manual, which is part of the supplementary package which can be saved to disk from VAST (under “Info/Save Documentation .ZIP To Disk …”) or downloaded from the VAST webpage^4^.

“VastTools.m” is a Matlab (The Mathworks, Inc.) script which can communicate with VAST via its API. It implements a number of supplementary functions, in particular for data exporting. VastTools is also included in VAST’s supplementary package. Since it is a Matlab script, its source code is fully readable and it can serve as a reference for implementing other client programs. Also, once VastTools is running and connected to VAST, other Matlab scripts can simply call API functions through the hidden global variable “vdata.vast”.

**FIGURE 6:**
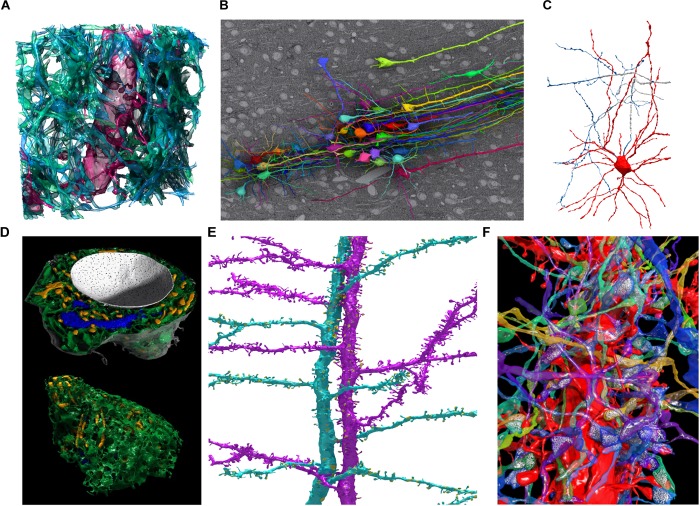
Examples of 3D models segmented in VAST and rendered in Autodesk 3ds Max. **(A)** Spiny dendrite (red) and axons (green) in a 10 μm × 10 μm × 6 μm cube of mouse cortex. At a voxel size of 6 nm × 6 nm × 30 nm, even the finest neural processes are segmentable. **(B)** Neurons traced in a low-resolution EM stack of mouse cortex, shown *in situ* above one EM section, with apical dendrites running in a bundle toward the pia (to the **right** in the image). Field of view roughly 510 × 340 micrometers. **(C)** Putative basket cell in rat cortex, traced semi-automatically with *trans*-layer masking in a ∼100 μm × 100 μm × 200 μm tissue volume. Tracing this cell took ∼15 h for a single expert. **(D)** Organelles in a neuron cell body (same cell as **Figure 5C**) shown from two directions. Nucleus white, with pores visible; endoplasmic reticulum green, mitochondria yellow, Golgi apparatus blue. **(E)** Two spiny apical dendrites with side branches in rat cortex. Synapses shown in yellow. **(F)** Spiny dendrite in red, with transparent axons making synapses on it. Neurotransmitter vesicles (white) were exported using particle clouds to generate spherical vesicles. All images are based on EM data from the lab of Jeff Lichtman, Harvard. All segmentations except **C** were done fully manually. **A,B,D,F** used data published with (Kasthuri et al., 2015).

The export functions of VastTools are summarized in **Table 2**. Most importantly, VastTools can export surface meshes of segmented objects to generic Wavefront OBJ files, which can then be imported into 3D rendering applications like 3ds Max (Autodesk, Inc.; see example images in **Figure 6**), Blender (Blender Foundation) or Unity (Unity Technologies). It can also export isosurface meshes (based on voxel brightness) which can for example be used to visualize fluorescence signals in light microscopic image stacks, and particle clouds, where a prototype 3D object (for example a small sphere representing a vesicle) is placed at the centers of all separately painted regions and the compound object is exported (3D object instancing). Furthermore, 3D boxes at specific locations in the data set can be exported as wireframe models or with single-color or textured sides, with a texture derived automatically from the loaded image stack. Finally, VastTools can export correctly sized scale bar models. **Figure 6** shows a selection of renderings made from 3D models labeled in and exported from VAST.

**FIGURE 7:**
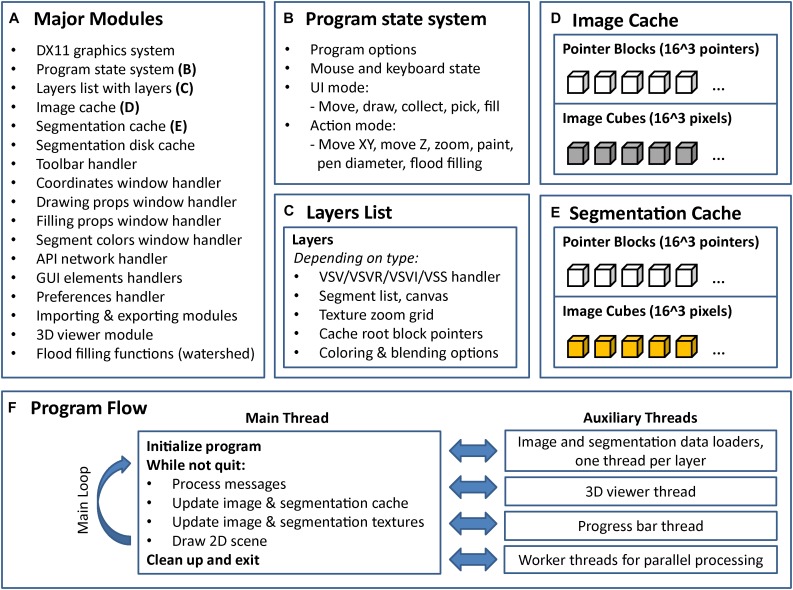
Internal program structure and control flow of VAST. VAST is structured as a collection of modules (C++ classes) which implement different parts of the program, the most important of which are listed in **(A)**. The internal state of the program is held in a global state system class **(B)**. Layers are kept in a linked list, with each layer holding a set of further class instances depending on type **(C)**. There are two caching systems, one for image data **(D)** and one for segmentation data **(E)**. All image layers share the image cache and all segmentation layers share the segmentation cache. **(F)** Shows the threading structure and the control flow of the main thread. Even though each layer has its own loader thread, cache updating is done in the main thread only to prevent multithreading problems.

VastTools can also render and export projection images along cardinal axes from the loaded data with multiple options, including illumination simulation. These projection images can not only be exported but also be used for single-image-click 3D navigation in VastTools’ “Simple Navigator Image” functionality.

“Target Lists” in VastTools can be used to create and organize annotated lists of particular points of interest in the data set loaded in VAST. Target coordinates are stored in the list together with a zoom level and the ID of the selected segment, and text descriptions can be added to each target. The corresponding 2D view of any listed target can then be restored in VAST with a single click in the target list.

Even though VAST is not a specialized analysis tool, several measurement functions are included in VastTools. First, Euclidian distances between any two points in the image stack can be measured. Second, VastTools can calculate the volumes of the volumetrically labeled segments and estimate their surface area during mesh exporting by summing the area of all generated mesh triangles for the object. This measurement is however likely to provide an overestimate of the true surface area because of the roughness of voxel models. A better estimate could be generated externally after smoothing the 3D models. Finally, VastTools can count the number of separately painted regions and export a list of centroid coordinates during particle cloud exporting.

The features discussed here are described in more detail in VASTs user manual.

## Discussion

Volume Annotation and Segmentation Tool is a light-weight and versatile tool specialized for volumetric annotation and segmentation of objects in very large image stacks. It is a self-contained program which is simple to set up and use even for inexperienced users. Typical applications include: Exploring very large EM and LM image stacks interactively; efficient manual segmentation of arbitrary structures to generate voxel training data for automatic segmentation algorithms; and sparse volumetric tracing of objects of interest, either manually or assisted by an automatic segmentation of the volume. VAST can also be used to proofread automatic segmentations, which is likely to become an important part of connectomic studies now that automated methods are becoming more commonplace.

Volume Annotation and Segmentation Tool is the only tool to our knowledge which can load multiple image and segmentation layers at the same time and use *trans*-layer masking to speed up manual segmentation and to proof-read automatic segmentations. For example, an automatic segmentation, even if it contains many errors, can be loaded as a separate layer and used to provide masks for painting objects with perfect outlines. Users can then volumetrically label neurites instantly while scrolling through the data set, while precise boundaries of the segmented objects are defined by the automatic segmentation. The 3D filling function speeds this process up further. Any defects caused by splits and mergers in the automatic segmentation can be corrected immediately by painting and filling. This process can also be seen as proof-reading a segmentation, in which original and proof-read versions are kept strictly separate because they reside in different layers.

In this way, volumetric voxel painting in VAST can reach speeds comparable to manual skeleton tracing based on placing nodes while scrolling through a 3D EM image stack. In both cases the speed is likely limited by either the rate at which images can be loaded and displayed, or by the speed at which humans can reliably follow a winding process through the neuropil. Voxel painting immediately generates filled-in areas of object cross-sections, which makes it easier for human observers to spot splits and mergers. Also, it natively produces volumetric objects, which for skeleton tracing requires an additional step. Also, different from tools which perform segmentation proof-reading by agglomerating object parts, in VAST proof-read segments are represented as voxels, which gives the user full freedom to modify them by painting as needed.

With VAST, these operations can be performed on image stacks which can reach Petabytes, including datasets which are streamed from a network source. As datasets grow larger and more and more research labs get interested in reusing these data-rich EM image volumes to address various scientific questions, online hosting and remote access will become more commonplace.

### Limitations

However, there are data sets and segmentation tasks for which VAST in its current form is not suitable. First of all, VAST was designed as a single-user standalone program, and as such it is not a multi-user client-server solution in which multiple annotators can simultaneously contribute to a shared server-hosted segmentation. While VAST allows image stacks to be loaded dynamically from a server, segmentation layers are currently hosted in local files only. It is possible to combine the segmentation results from multiple users by merging segmentation files, but this is comparatively slow and inconvenient. Therefore it is tricky to use VAST in settings where numerous annotators are working together to segment or proof-read the same segmentation volume. A future version may implement a segmentation layer type in which the data is hosted on and synchronized with a server, for interactive multi-user editing. The server would also keep track of the changes each user makes (provenance tracking and version control), which becomes more important in multi-user settings. In VAST, provenance can be tracked rudimentarily by saving to a new file every time and keeping the old versions, and having different users keep their work separate.

Second, as a voxel painting program, VAST is not equipped to work with skeletons or surface mesh data directly. It is possible to use voxel painting to indicate skeleton lines of 3D objects, and to analyze these externally to generate skeletons (Morgan et al., 2016), but this is more a work-around than a true solution. VAST does not use surface meshes internally either; for example the 3D viewer uses a three-dimensional texture instead. Surface meshes of segmented objects can however be generated in VastTools for external analysis and rendering. Measurement of lengths, diameters, axonal branching patterns etc. are also not included in VAST but have to be performed externally.

When using skeletons, synapses between two neurons can be represented by edges of a special ‘synapse’ type, bridging between the skeletons of two cells. This is not possible in VAST since it does not provide skeletons. However, voxel annotation of synapses can be (and has been) used to compute synaptic connectivity (Kasthuri et al., 2015; Morgan et al., 2016; Quadrato et al., 2017). If synapses are manually or automatically labeled so that the synapse region overlaps with the pre- and postsynaptic partner neurites volumetrically, the synapse label can then be used as a mask to extract the IDs of the connected neurites automatically. This can be done with an external script which uses either the API or exported segmentation image stacks.

While VAST has no problem allowing users to label all the synapses in a dataset, it is not a tool specialized for the analysis of connectivity structure or other more sophisticated analyses of tissue morphology. Data on synaptic partners can be exported and analyzed downstream with specific tools, as was done in (Kasthuri et al., 2015; Morgan et al., 2016; Quadrato et al., 2017). The possible future addition of explicit skeletons associated with the volumetric labels in VAST may make many of these tasks more straight-forward.

Currently, the 2D view in VAST is limited to XY sections, mainly because of our anisotropic ATUM data sets and the fact that using only XY mip maps saves time and storage space. Data can in principle be resliced externally if tracing at a different orientation is preferable. However, supporting different mip mapping and 2D slicing options, as well as improved capabilities of the 3D viewer, may be useful features in the future.

Finally, VAST is based on the Windows user interface and graphics system (Direct3D 11) for speed and simplicity, and cannot easily be ported to other operating systems. Also it is currently not an open source program, and feature additions and bug fixes depend on the developers. However, its API is fully documented and many functions can be accessed remotely for external processing and script-based automation to add custom functionality.

Though VAST is still being developed further and more features are added as needed, its main strength is manual and semiautomatic segmentation by voxel painting. Extensive import and export functions are provided, including an API, so that VAST can play its role as a powerful tool in a larger pipeline.

## Data Availability Statement

No datasets were generated for this study. At the time of writing of this manuscript, the current version of VAST can be downloaded at: https://software.rc.fas.harvard.edu/lichtman/vast/

## Author Contributions

DB wrote VAST. HS and JL supported the development and provided advice and feedback. DB and JL wrote the manuscript with input from HS.

## Conflict of Interest Statement

The authors declare that the research was conducted in the absence of any commercial or financial relationships that could be construed as a potential conflict of interest.

## Footnotes

1 https://github.com/janelia-flyem/NeuTu

2 http://www.youtube.com/watch?v=BZ_0TVMSdjA

3 http://www.youtube.com/watch?v=4XCNRgDzSjc

4 http://software.rc.fas.harvard.edu/lichtman/vast/
